# Ranolazine Improves Glycemic Variability and Endothelial Function in Patients with Diabetes and Chronic Coronary Syndromes: Results from an Experimental Study

**DOI:** 10.1155/2021/4952447

**Published:** 2021-12-31

**Authors:** Annunziata Nusca, Federico Bernardini, Fabio Mangiacapra, Ernesto Maddaloni, Rosetta Melfi, Elisabetta Ricottini, Francesco Piccirillo, Silvia Manfrini, Gian Paolo Ussia, Francesco Grigioni

**Affiliations:** ^1^Unit of Cardiac Sciences, Department of Medicine, Campus Bio-Medico University of Rome, Via Alvaro del Portillo 200, 00128 Rome, Italy; ^2^Department of Experimental Medicine, Sapienza University of Rome, Viale Regina Elena 324, 00161 Rome, Italy; ^3^Unit of Endocrinology and Diabetes, Department of Medicine, Campus Bio-Medico University of Rome, Via Alvaro del Portillo 200, 00128 Rome, Italy

## Abstract

**Background:**

Ranolazine is a second-line drug for the management of chronic coronary syndromes (CCS). Glucose-lowering and endothelial effects have also been reported with this agent. However, whether ranolazine may improve short-term glycemic variability (GV), strictly related to the prognosis of patients with type 2 diabetes (T2D), is unknown. Thus, we aimed to explore the effects of adding ranolazine to standard anti-ischemic and glucose-lowering therapy on long- and short-term GV as well as on endothelial function and oxidative stress in patients with T2D and CCS.

**Methods:**

Patients starting ranolazine (*n* = 16) were evaluated for short-term GV, haemoglobin 1Ac (Hb1Ac) levels, endothelial-dependent flow-mediated vasodilation (FMD), and oxidative stress levels at enrolment and after 3-month follow-up. The same measurements were collected from 16 patients with CCS and T2D that did not receive ranolazine, matched for age, gender, and body mass index.

**Results:**

A significant decline in Hb1Ac levels was reported after 3-month ranolazine treatment (mean change -0.60%; 2-way ANOVA *p* = 0.025). Moreover, among patients receiving ranolazine, short-term GV indexes were significantly improved over time compared with baseline (*p* = 0.001 for time in range; 2-way ANOVA *p* = 0.010). Conversely, no significant changes were reported in patients without ranolazine. Finally, greater FMD and lower oxidative stress levels were observed in patients on ranolazine at 3 months.

**Conclusions:**

Ranolazine added to standard anti-ischemic and glucose-lowering therapy demonstrated benefit in improving the glycemic status of patients with T2D and CCS. How this improvement contributes to the overall myocardial benefit of ranolazine requires further studies.

## 1. Background

Ranolazine mainly exerts its anti-ischemic effects by inhibiting the myocardial late sodium current [1]. According to guidelines, it is approved as a second-line treatment in managing patients with chronic coronary syndromes (CCS) inadequately controlled by or intolerant to first-line anti-ischemic agents [2]. However, the blockage of late sodium channels by this drug has also been reported in other tissues such as pancreatic islets [3]. Hence, ranolazine significantly lowered fasting plasma glucose (FPG) and haemoglobin A1c (HbA1c) levels in diabetic patients naïve to previous antidiabetic medications or already on glucose-lowering agents at least twelve weeks of treatment later, suggesting a specific role of this drug in patients with the coexistence of CCS and type 2 diabetes (T2D) [4–6].

While the hypothesis that ranolazine may simultaneously improve myocardial ischemia and glycemic control in patients with T2D and CCS is intriguing, most of the available data in this regard derives from post hoc analyses of studies not specifically designed to explore this issue, using high dose ranolazine formulations (750-1000 mg) [4–6]. Moreover, most of these investigations focused on changes in FPG and Hb1Ac levels, parameters that may offer an incomplete assessment of glucose control in T2D patients. Conversely, short-term glycemic variability (GV) includes an overall measure of intraday and between-day glucose fluctuations. Interestingly, GV demonstrated to add significant information, on top of HbA1c values, for the prediction of micro- and macrovascular complications, hypoglycemic episodes, and all-cause mortality in T2D patients [7–10]. Currently, no study has specifically investigated the effects of ranolazine on short-term GV.

Thus, in this experimental study, we primarily aimed to explore the effects of adding ranolazine to standard anti-ischemic and glucose-lowering medical therapy on patients' glycemic control, assessed by FPG, Hb1Ac, and short-term GV indexes, in a cohort with T2D and CCS. Moreover, we secondarily investigated the impact of ranolazine on endothelial function and oxidative stress.

## 2. Methods

Between March and June 2016, we prospectively enrolled at our institution 16 patients with an established diagnosis of T2D and CCS receiving ranolazine according to guidelines (375 mg twice a day for the first 4 weeks, with a subsequent upgrading to 500 mg and then to 750 mg twice a day, depending on the patient's response and tolerance). The decision concerning ranolazine initiation and subsequent dosage escalation was left to the treating physicians.

Exclusion criteria were type 1 diabetes or secondary forms of diabetes; acute coronary syndromes in the previous month; severe renal failure (serum creatinine >1.8 mg/dl or glomerular filtration rate < 30 ml/min/1.73 m^2^); coexistent immunological, inflammatory, or neoplastic disease at the time of enrolment; known intolerance to ranolazine; QTc interval > 500 msec at baseline ECG or a personal or family history of QTc prolongation or use of drugs that prolong the QTc interval; concomitant treatment with CYP34A inhibitors; and unavailability of a glucose sensor for research purpose.

All patients were evaluated for GV, FPG, and Hb1Ac levels, endothelial-dependent flow-mediated vasodilation (FMD) of the brachial artery, and oxidative stress levels at enrolment and after a 3-month follow-up. For descriptive purposes, the same measures were collected from another 16 patients with CCS and T2D that did not receive ranolazine therapy at the same time interval, matched for age, gender, and body mass index.

The study was conducted following institutional guidelines, national and local legal requirements, and the Declaration of Helsinki. The research protocol was approved by our local Ethical Committee (study reference number-02.16 TS.ComETCBM), and all patients gave their informed consent. We respected the Strengthening the Reporting of Observational Studies in Epidemiology (STROBE) guidelines to conduct the study.

### 2.1. Glycemic Variability Assessment

Samples were collected for Hb1Ac and FPG measurements at baseline and after a three-month follow-up. Short-term GV was assessed using the iPro™ continuous glucose recorder (Medtronic, Northridge, CA), as previously described [10]. Thus, a continuous glucose monitoring (CGM) sensor (Enlite® Sensor) was inserted into the patients' subcutaneous abdominal fat tissue for a 24-hour interval; this device was able to measure subcutaneous tissue interstitial glucose levels every 5 minutes, within a range of 40-400 mg/dl. During iPro™ CGM, patients also checked their capillary blood glucose levels with self-monitoring at least 4 times per day. The FreeStyleLite (Abbott Laboratories, Abbott Park, IL) BG-monitoring system was used to calibrate the iPro™ continuous glucose recorder. After 24-hour monitoring, the recorded data were downloaded for analysis through the CareLinkiPro System. The following parameters of ambulatory glucose metrics were calculated according to the international consensus on time in range (TIR) [11]: TIR, as the percentage of time spent with blood glucose values between 70 and 180 mg/dl; time below range (TBR), as the percentage of time spent with blood glucose values below 70 mg/dl; and time above range (TAR), as the percentage of time spent with blood glucose values above 180 mg/dl. The following indexes of intraday GV were also calculated as previously explained [7]: standard deviation (SD), coefficient of variation (CV), and mean amplitude of glycemic excursions (MAGE).

### 2.2. Flow-Mediated Vasodilation

FMD was assessed according to the updated expert consensus' guidelines for flow-mediated dilation in humans [12]. Patients were maintained fasted (>6 h), and all vasoactive drugs were discontinued for at least 48 hours before the measurement. A first longitudinal scan of the artery (a straight segment of 8 to 10 mm in length) was selected above the antecubital fossa for measurements (baseline values) using a 10 MHz linear probe. Afterwards, a blood pressure cuff was inflated on the same upper arm to 50 mmHg above the patient's systolic pressure for 5 minutes and then deflated. After one minute, a second longitudinal scan was obtained to recalculate the brachial artery diameter (postocclusion values). Measurements were made at the end of the diastole, corresponding with the R wave on a continuously recorded electrocardiogram; moreover, five cardiac cycles were consecutively analyzed and averaged for each scan. Thus, FMD, due to shear-induced endothelial nitric oxide release, was expressed as percent diameter variation. Intraobserver variation was calculated to be 1.02 ± 0.5 in 10 randomly selected patients undergoing FMD assessment.

### 2.3. Measurement of Derivative Reactive Oxygen Metabolites

The derivative reactive oxygen metabolite (d-ROM) test measured the concentration of hydroperoxides (ROOH) using the FRAS-4 Evolvo system (H&D, Parma, Italy) [13]. After blood centrifugation at 3000 rpm for 10 min at 4°C, fresh serum was mixed with an acidic medium, and the transition metal ions formed catalyzed the hydroperoxide breakdown, generating alkoxy and peroxyl radicals (reactive oxygen species, ROS). Afterwards, by adding a chromogen (N,N-diethyl-paraphenylenediamine), the number of hydroperoxides produced was quantified by detecting the density of the colored complex with a dedicated analytical photometric device. One unit of d-ROMs (U.CARR (Carratelli units)) corresponds to 0.8 mg/l hydrogen peroxide [14], and values from 250 to 300 U.CARR. were defined as the normal range. The intra- and interassay coefficients of variation were 0.3–6.6% and 0.3–5.1%, respectively [15]. By contrast, the plasma antioxidant test (PAT) was used to detect the biological antioxidant potential of patients' blood plasma by measuring the capacity to reduce iron from the ferric (Fe3+) to ferrous form (Fe2+). The chromatic change of this reaction was measured photometrically. The results were expressed in U.Cor., where 1 U.Cor. = 1.4 *μ*mol/l of vitamin C. The normal range varied between 2200 and 2800 U.Cor. [16].

### 2.4. Statistical Analysis

A sample size of 16 patients in the ranolazine group was calculated to provide the study with 90% power at a significance level of 0.05 to show a clinically meaningful 30% change of TIR from baseline, with an estimated standard deviation of 15%. Data are expressed as frequencies and percentages for categorical variables and mean ± standard deviation or median [Q1, Q3] for continuous variables. The Shapiro-Wilk test was used to identify deviations from the normal distribution. A paired *t*-test or Wilcoxon signed-rank test was applied to investigate differences among means (or medians) between baseline and 3-month follow-up of paired parametric and nonparametric continuous values. Differences between groups (ranolazine and no ranolazine) in parametric and nonparametric continuous variables were tested with the Student *t*-test and Mann–Whitney *U* test. Fisher exact test or Pearson *χ*^2^ test was used to compare categorical variables. A two-way ANOVA for repeated measures followed by pairwise comparisons was used to detect changes in GV indexes over time in the study groups. A two-tailed *p* value < 0.05 was considered statistically significant. The statistical analyses were performed using SPSS version 20.

## 3. Results

Baseline demographical and clinical characteristics of enrolled patients are described in Table 1. As expected, patients receiving ranolazine more frequently reported symptoms of angina classes III-IV according to the Canadian Cardiovascular Society classification and had a better renal function than those who did not receive the drug. All patients in both groups had a Hb1Ac > 5.7%; similarly, most enrolled patients had a baseline FPG value > 100 mg/dl (75% of those receiving ranolazine and 62% in the placebo group).

Of note, ranolazine was started in all patients at 375 mg twice daily; after four weeks in 9 (56%) patients, the ranolazine dosage was increased to 500 mg twice daily for symptoms persistence; none of the patients required up-titration to 750 mg twice daily or down-titration/drug discontinuation for clinical or ECG intolerability. Moreover, during the study period, no changes in antidiabetic therapy were applied to patients with and without ranolazine.

### 3.1. Glycemic Variability

**Figure 1:**
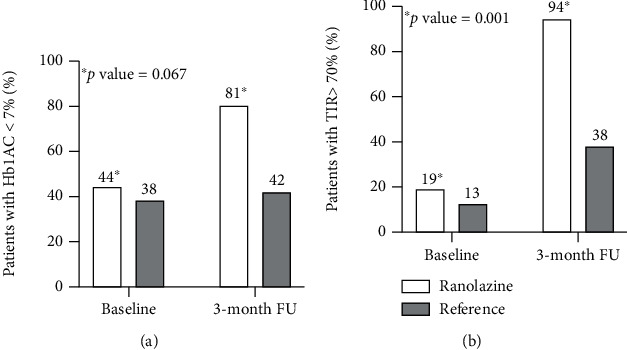
Ranolazine effect on Hb1Ac and short-term GV. (a) Patients with Hb1Ac < 7% at baseline and 3 months in the two groups; (b) Patients with TIR > 70% at baseline and 3 months in the two groups. Hb1Ac: haemoglobin 1Ac; TIR: time in range.

Table 2 reports glycemic parameters measured in the two cohorts at enrolment and after follow-up. At three months, patients on ranolazine showed lower Hb1Ac and FPG levels compared with baseline (*p* < 0.001 for Hb1Ac, mean change -0.60%; *p* = 0.072 for FPG). Two-way ANOVA analysis confirmed the meaningful decline in Hb1Ac levels over time in patients receiving the drug compared to those who did not (*p* = 0.025). A higher percentage of patients achieved the Hb1Ac target < 7% during follow-up among the ranolazine cohort (81 versus 44% at baseline, *p* = 0.067) (Figure 1(a)).

Accordingly, patients treated with ranolazine showed a significant increase in TIR values at 3-month follow-up [78 (73, 96) versus 61 (46, 74) at baseline, *p* = 0.001] (2-way ANOVA comparing to patients without ranolazine, *p* = 0.010). At this time, in the ranolazine group, a TIR value > 70% was obtained in 94% of patients than 19% at baseline (*p* < 0.001); differently, 13% and 38% of patients who did not receive the drug reported a TIR value > 70% at baseline and 3-month follow-up, respectively (*p* = 0.102) (Figure 1(b)). A significant reduction in TAR (*p* = 0.010) and a trend towards a lower TBR (*p* = 0.116) were also observed among the ranolazine cohort over time.

Furthermore, ranolazine therapy was associated with a significant decline in all other short-term GV indexes; thus SD, CV, and MAGE (*p* = 0.023, *p* = 0.020, and *p* = 0.042 compared with pretreatment values, respectively) (Table 2). Contrariwise, no significant improvement was observed in patients not receiving ranolazine.

### 3.2. Flow-Mediated Dilation and Oxidative Stress

During follow-up, ranolazine treatment resulted in a significant FMD increase compared to baseline (*p* = 0.039) (Table 3), so how significantly fewer patients on ranolazine showed a persistent FMD value < 7% at 3 months (6% versus 44% at baseline, *p* = 0.014).

Patients receiving ranolazine exhibited a marked but not significant reduction in d-ROMs from baseline to 3-month follow-up (*p* = 0.075). Likewise, the antioxidant activity assessed by the PAT test was significantly increased in those patients during follow-up (*p* = 0.007 compared with baseline) (Table 3). No changes were reported from baseline to 3 months among patients who did not receive ranolazine.

**Figure 2:**
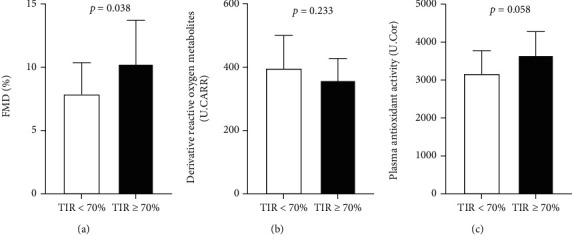
Impact of glycemic control on endothelial function and oxidative stress. FMD (a), d-ROMs (b), and PAT (c) values in patients with and without TIR > 70% at 3 months in the overall population (*n* = 32). FMD: flow-mediated dilation; d-ROMs: derivative reactive oxygen metabolites; PAT: plasma antioxidant test.

As expected, patients with a better glycemic control after three months, defined by a TIR > 70%, revealed significantly greater FMD and a trend toward lower d-ROMs and higher PAT levels (Figure 2).

## 4. Discussion

To our knowledge, this is the first study focused on the effects of ranolazine treatment on both short-term and long-term GV indexes, reporting a significant improvement of glycemic control in patients with T2D and CCS receiving this drug. We also confirmed the favorable properties of ranolazine on endothelial dysfunction and oxidative stress. Interestingly, ranolazine benefit was revealed on top of vasoactive and anti-inflammatory cardiovascular therapies (ACE-inhibitors and statins), despite adequate glucose-lowering treatment.

### 4.1. Effects of Ranolazine on Haemoglobin 1Ac

In our study, ranolazine was associated with a significant Hb1Ac decrease after a 3-month (12-week) treatment (absolute difference of 0.60%). These findings confirm previous data from post hoc analyses of large, randomized trials showing an absolute decrease of 0.48% and 0.64% in Hb1Ac with ranolazine after 12-16 weeks [4, 17]. Similarly, the only trial specifically designed to investigate the impact of ranolazine on glycemic control in T2D patients revealed a noticeable Hb1Ac reduction of 0.76% after 24-week treatment [6]. However, in these previous investigations, ranolazine ameliorated glycemic status in a dose-dependent manner, thus with a greater benefit in patients receiving high ranolazine dosages (750 or 1000 mg) and in those with poorer glycemic control or on insulin therapy [4, 17, 18]. Of note, in our study, ranolazine was already effective at lower doses (with only 56% of patients on 500 mg twice daily) and in patients with a moderate glycemic control at baseline (mean Hb1Ac 7.2 ± 0.7%). Previously, only another small study reported improved Hb1Ac levels with 500 mg ranolazine twice daily [19].

### 4.2. Effects of Ranolazine on Glycemic Variability

Short-term GV assessed by CGM provides a more comprehensive and meaningful measure of glycemic status than traditional glycemic parameters such as Hb1Ac and FPG [7, 8, 11]. The major advantage of short-term GV is that it includes all glycemic changes in a specific time interval, thus considering also hypoglycemic episodes, prognostically relevant in patients with cardiovascular diseases [20]. However, no previous study investigated the effects of ranolazine on GV.

We reported a significant increase of TIR values among patients treated with ranolazine from baseline to 3-month follow-up. TIR, expressing the time that a patient spends within an optimal glycemic range (70-180 mg/dl), has recently emerged as the most accurate metric of short-term GV [8, 11]. It has been inversely associated with overall and cardiovascular mortality and the development of microvascular complications in patients with T2D [21, 22]. Likewise, MAGE demonstrated to be an independent predictor of poor prognosis in diabetic patients with acute and chronic coronary syndromes [23], having been associated with the development of macrovascular complications and increased platelet reactivity [9, 10]. Of note, according to our data, ranolazine exerts its lowering effects also on this latter GV measurement.

### 4.3. Clinical Implications

Myocardial ischemia causing angina is promoted by the presence of obstructive coronary plaques and/or coronary microvascular dysfunction. This last condition, characterized by impaired vasodilation in response to increased myocardial oxygen demand, has been reported to have a key role in the development of myocardial ischemia in diabetic patients [24]. Both sustained hyperglycemia and increased GV showed worse oxidative stress and inflammation, inducing coronary vasomotion abnormalities through the impairment of both endothelium-dependent and endothelium-independent vasodilation [25]. Furthermore, increased oxidative stress, together with the impairment of endogenous antioxidant mechanisms, has been reported to cause abnormal ion channels' function in cardiomyocytes and endothelial cells [25].

**Figure 3:**
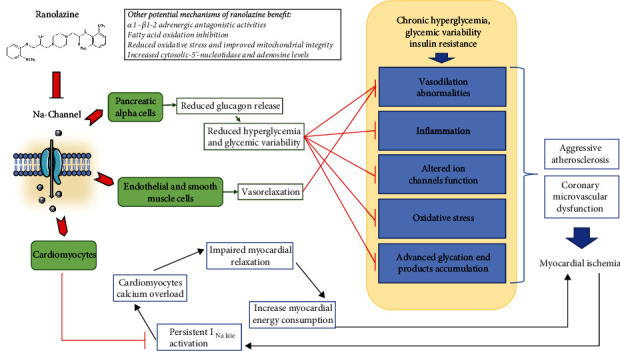
Mechanisms of ranolazine's benefit in the diabetic patient.

In this pathophysiological scenario, ranolazine may exert significant positive actions mediated by the blockage of the late sodium channel current (Figure 3). In myocardiocytes, ranolazine may protect against ischemia by reducing cytosolic and mitochondrial calcium overload and improving mitochondrial integrity [26]. By blocking sodium-channel isoform Na_Y_1.3 in pancreatic alpha cells [3], it inhibits glucagon release, thus reducing hyperglycemia and glycemic variability. Of note, other ranolazine-mediated glycometabolic effects have been described; thus, this drug demonstrated to preserve the morphology and function of pancreatic beta cells [27] and, in a rat model, to improve glycemic control by expanding muscle microvascular endothelial surface area and increasing insulin delivery and action [28]. Finally, ranolazine's direct inhibitory effect on endothelial cell sodium channels and its anti-inflammatory and antioxidative actions may contribute to the improvement in flow-mediated dilation observed in our study as well as in previous investigations [29]. Indeed, we found a significant enhancement in endothelial function after three months, along with a decrease in oxidative stress markers and increased antioxidant activity in patients receiving ranolazine. Significantly, we could speculate that the ranolazine benefit on the myocardium and endothelium may be potentially amplified by its glucose-lowering effect given the deleterious impact of hyperglycemia on endothelial function; however, dedicated experimental studies are needed to confirm this hypothesis. In this regard, the anti-ischemic benefit of ranolazine seems to be even more evident in diabetic patients with abnormal glycemic control; actually, the River PCI trial showed a further reduction in angina frequency in patients with poor glycemic control (Hb1Ac ≥ 7.5%) compared with those with Hb1Ac levels below this threshold [30].

Furthermore, diabetes promotes the development and destabilization of coronary atherosclerosis. Plaques from patients with T2D have demonstrated a higher inflammatory burden and macrophage accumulation [31]. Accordingly, diabetic patients who experienced acute coronary syndromes have poor prognoses than those without diabetes [32, 33]. In this scenario, some antidiabetic agents such as metformin and incretins demonstrated anti-inflammatory properties beyond their glucose-lowering effects, improving endothelial function, and reducing cardiac adverse events [34–36]. According to our results, ranolazine might potentially strengthen the antioxidative actions of these antidiabetic agents in preventing plaque progression and adverse cardiac event occurrence.

Finally, the use of ranolazine in patients with T2D and CCS could be extremely useful in the framework of a multifactorial therapeutic approach [37], improving symptoms and exercise tolerance on the one hand and, on the other, optimizing glycemic control and endothelial function. Notably, glycemic control significantly impacts the cardiovascular prognosis of diabetic patients [21, 38, 39]. Otherwise, randomized trials investigating the anti-ischemic effects of ranolazine did not report any benefit in terms of clinical endpoints such as cardiovascular death or acute cardiac events in patients with CCS regardless of the presence of T2D [40, 41]. Given these conflicting results, further studies are warranted to investigate the potential prognostic benefit of ranolazine in the subset of diabetic patients achieving an improved glycemic control with the addition of this drug.

### 4.4. Study Limitations

The study is limited by the observational design and the small sample size. Moreover, GV assessment using continuous glucose monitoring was performed for 24 hours, with a probably less accurate collection of glycemic data than a longer interval as suggested by a recent international consensus [11]. However, this is the first study investigating the effects of ranolazine on GV, and even though a more extended period of monitoring might translate into a more precise GV evaluation, it would be less applicable to current clinical practice. The causality between ranolazine treatment and improved glycemic control, as well as increased FMD or reduced oxidative stress, can only be inferred. Furthermore, our sample size did not allow us to investigate the additional potential benefit of ranolazine on each antidiabetic agent. Furthermore, we did not investigate the potential metabolic effect of ranolazine in patients with prediabetes or normoglycemia, where antidiabetic agents have still demonstrated anti-inflammatory effects [34, 35]. By the way, we wanted to investigate the additional potential benefit of ranolazine on top of anti-ischemic and glucose-lowering therapies in diabetic patients with CCS. Lastly, we did not report data on symptoms and quality of life; however, this was not our purpose since previous large trials reported effective ranolazine anti-ischemic effects.

## 5. Conclusions

Ranolazine added to standard anti-ischemic and glucose-lowering therapy demonstrated benefit in improving glycemic status, assessed by Hb1Ac and short-term GV indexes, and endothelial function in a cohort of patients with T2D and CCS. These findings may endorse the specific role of this drug in diabetic patients with cardiovascular disease, reinforcing its use in a tailored medical therapy approach whereby other therapeutic invasive options have reported lower efficacy.

## Figures and Tables

**Table 1:** Baseline characteristics of the two cohorts.

	Ranolazine (*n* = 16)	No ranolazine (*n* = 16)	*p* value
Age (years)	69 ± 8	72 ± 8	0.268
Female gender	4 (25)	4 (25)	—
BMI (kg/m^2^)	30 ± 5	29 ± 4	0.224
Dyslipidemia	14 (88)	12 (75)	0.365
Hypertension	15 (94)	15 (94)	—
Smoking	10 (63)	11 (69)	0.709
Diabetes on oral GLAs	12 (75)	8 (50)	0.273
Diabetes on insulin therapy	3(19)	5 (31)	0.685
Diabetes on oral GLAs and insulin	1 (6)	3 (19)	0.597
Previous PCI	12 (75)	11(69)	0.694
Previous CABG	3 (19)	3 (19)	—
LVEF (%)	56 ± 7	50 ± 9	0.089
Angina (CCS III-IV)	5 (31)	1 (6)	0.172
Multivessel disease	12 (75)	11 (69)	0.694
Haemoglobin (g/dl)	13.6 ± 1.2	13.0 ± 1.9	0.375
Creatinine (mg/dl)	0.9 ± 0.2	1.1 ± 0.3	0.086
GFR (ml/min/1.73 m^2^)	82.0 ± 26.5	73.6 ± 24.3	0.364
Statins	14 (88)	15 (94)	0.544
Beta-blockers	12 (75)	12 (75)	—
ACE-inhibitors/ARBs	13 (81)	16 (100)	—
Nitrates	10(63)	11 (69)	0.709
Calcium-channel blockers	8 (50)	11 (69)	0.472
Biguanides	11 (69)	9 (56)	0.716
Sulfonylureas	5 (31)	1 (6)	0.172
Meglitinides	3(19)	2 (13)	1
TZD	0	0	—
DPP-4 inhibitors	3 (19)	2 (13)	1
SGLT-2 inhibitors	0	1 (6)	—
GLP-1 agonists	0	0	—
Insulin	4 (25)	8 (50)	0.273

Values are mean ± SD or *n* (%). ACE: angiotensin-converting enzyme; ARB: angiotensin receptor blockers; BMI: body mass index; CABG: coronary artery bypass surgery; CCS: Canadian Cardiovascular Society; DPP: 4-dipeptidyl-peptidase 4; GLA: glucose-lowering agents; GLP-1: glucagon-like peptide 1; GFR: glomerular filtration rate; LVEF: left ventricular ejection fraction; PCI: percutaneous coronary intervention; SGLT2: sodium-glucose transporter 2; TZD: thiazolidinediones.

**Table 2:** Glycemic variability at baseline and after three months.

	Ranolazine			No ranolazine		
	Baseline	3 months	*p* value	Baseline	3 months	*p* value
FPG (mg/dl)	121 ± 36	102 ± 14	0.072	131 ± 61	119 ± 28^∗^	0.460
Hb1Ac (%)	7.2 ± 0.7	6.6 ± 0.5	<0.001	7.6 ± 1.2	7.5 ± 1.1^∗^	0.928
TIR (%)	61 (46, 74)	78 (73, 96)	0.001	51 (28, 71)	62(15, 77)^∗^	0.408
TBR (%)	0 (0, 13)	0 (0, 0)	0.116	0 (0, 9)	0.5 (0.5, 10)	0.929
TAR (%)	28 (10, 42)	16 (4, 26)	0.010	40 (24, 67)	33 (10, 85)	0.277
SD (mg/dl)	32.7 ± 11.1	25.3 ± 9.2	0.023	37.3 ± 13.0	33.0 ± 14.7	0.269
CV (%)	28.0 ± 9.0	21.2 ± 8.3	0.020	27.6 ± 10.8	23.8 ± 7.4	0.186
MAGE (mg/dl)	56.8 ± 23.6	44.8 ± 18.1	0.042	56.3 ± 18.1	58.8 ± 17.2^∗^	0.653

^∗^ *p* < 0.05 compared with 3-month results in the ranolazine cohort; values are mean ± SD or median(Q1, Q3). CV: coefficient of variation; FPG: fasting plasma glucose; HbA1c: haemoglobin A1c; MAGE: mean amplitude glycemic excursions; SD: standard deviation; TAR: time above range; TBR: time below range; TIR: time in range.

**Table 3:** Flow-mediated dilation and oxidative stress at baseline and after 3-month follow-up.

	Ranolazine			No ranolazine		
	Baseline	3 months	*p* value	Baseline	3 months	*p* value
Baseline artery diameter (cm)	0.45 ± 0.10	0.49 ± 0.11	0.008	0.43 ± 0.06	0.44 ± 0.06	0.411
Hyperemia-induced diameter (cm)	0.49 ± 0.09	0.53 ± 0.11	<0.001	0.46 ± 0.06	0.47 ± 0.07	0.280
FMD (%)	8.7 ± 4.4	11.1 ± 3.3	0.039	7.1 ± 3.0	7.8 ± 2.5^∗^	0.445
d-ROMs (U.CARR)	407 ± 90	358 ± 71	0.075	377 ± 63	386 ± 93	0.647
PAT (U.Cor.)	3255 ± 282	3715 ± 628	0.007	3284 ± 653	3247 ± 601	0.888

^∗^ *p* < 0.05 compared with 3-month results in the ranolazine cohort; Values are mean ± SD. FMD: flow-mediated dilation; d-ROMs: derivative reactive oxygen metabolites; PAT: plasma antioxidant test.

## Data Availability

The authors declare that all data supporting the findings of this study are available within the article. The datasets used during the current study are available from the corresponding author on reasonable request.

## Abbreviations

CCS: Chronic coronary syndromes
CV: Coefficient of variation
d-ROMs: Derivative reactive oxygen metabolites
FMD: Flow-mediated vasodilation
FPG: Fasting plasma glucose
GCM: Continuous glucose monitoring
GV: Glycemic variability
Hb1Ac: Haemoglobin 1Ac
MAGE: Mean amplitude of glycemic excursions
PAT: Plasma antioxidant test
ROS: Reactive oxygen species
SD: Standard deviation
T2D: Type 2 diabetes
TAR: Time above range
TBR: Time below range
TIR: Time in range.
